# End‐user involvement in a systematic review of quantitative and qualitative research of non‐pharmacological interventions for attention deficit hyperactivity disorder delivered in school settings: reflections on the impacts and challenges

**DOI:** 10.1111/hex.12400

**Published:** 2015-09-21

**Authors:** Jo Thompson Coon, Ruth Gwernan‐Jones, Darren Moore, Michelle Richardson, Catherine Shotton, Will Pritchard, Christopher Morris, Ken Stein, Tamsin Ford

**Affiliations:** ^1^ NIHR CLAHRC South West Peninsula (PenCLAHRC)University of ExeterExeterUK; ^2^ Third Gap Research GroupUniversity of ExeterExeterUK; ^3^ Health Service and Population Research DepartmentKings College LondonLondonUK; ^4^ Child Health GroupUniversity of Exeter Medical SchoolUniversity of ExeterExeterUK; ^5^ Babcock LDPExeterUK; ^6^ Peninsula Cerebra Research Unit (PenCRU)ExeterUK

**Keywords:** end‐user involvement, engagement, methods, systematic review

## Abstract

**Background:**

The benefits of end‐user involvement in health‐care research are widely recognized by research agencies. There are few published evaluations of end‐user involvement in systematic reviews.

**Objectives:**

(i) Describe end‐user involvement in a complex mixed‐methods systematic review of ADHD in schools, (ii) reflect on the impact of end‐user involvement, (iii) highlight challenges and benefits experienced and (iv) provide suggestions to inform future involvement.

**Methods:**

End‐users were involved in all stages of the project, both as authors and as members of an advisory group. In addition, several events were held with groups of relevant end‐users during the project.

**Results:**

End‐user input (i) guided the direction of the research, (ii) contributed to a typology of interventions and outcomes, (iii) contributed to the direction of data analysis and (iv) contributed to the robustness of the syntheses by demonstrating the alignment of interim findings with lived experiences. Challenges included (i) managing expectations, (ii) managing the intensity of emotion, (iii) ensuring that involvement was fruitful for all not just the researcher, (iv) our capacity to communicate and manage the process and (v) engendering a sense of involvement amongst end‐users.

**Conclusions:**

End‐user involvement was an important aspect of this project. To minimize challenges in future projects, a recognition by the project management team and the funding provider that end‐user involvement even in evidence synthesis projects is resource intensive is essential to allow appropriate allocation of time and resources for meaningful engagement.

## Introduction

There are compelling moral and ethical arguments for public involvement in research^1^, ^2^ and involvement is a requirement of many funding agencies.^3^, ^4^, ^5^ There are also assumptions that research in which the public has been involved will (i) be more credible and applicable and (ii) may be more readily translated into practice.^6^ Although NIHR INVOLVE define public involvement as involvement of patients, potential patients, carers and people who use health and social care services as well as people from organizations that represent people who use services,^5^ we feel that in order to enhance both the credibility of the research *and* the opportunities for findings to be translated into practice, it is also important to include those with a professional role in health and social care service who might realistically be expected to use the review findings in their practice. We therefore define end‐users as all those for whom the original research question is pertinent, and for this project, that included the families of children and young people with ADHD; teaching, special educational needs and mental health services professionals; researchers; charities involved in disseminating research findings; and those involved in the development and delivery of non‐pharmacological interventions for ADHD in the school setting.

Developing expertise in end‐user involvement in evidence synthesis may be particularly challenging. Many reviews are completed by groups of methodologists who are not necessarily topic experts and are unlikely to have topic‐specific connections with relevant end‐users. As systematic reviews are often considered to be the highest level of evidence, it would appear prudent to develop guidelines for best practice in involving end‐users in their conduct.

We recently completed and published a large evidence synthesis consisting of four systematic reviews including 138 studies related to non‐pharmacological interventions for ADHD used in school settings. The reviews considered the effectiveness of school‐based interventions for ADHD, attitudes towards and experience of school‐based interventions for ADHD, and the experience of ADHD in school settings. The findings of these reviews are published elsewhere.^7^ As part of the review process, we incorporated the perspectives of a number of potential end‐users of the review findings, for example parents and carers of children and young people with ADHD, teaching professionals and researchers. Although we and our funding body understand the importance of end‐user involvement and indeed the funding body require end‐user involvement in all projects, it was not the focus of the project, and thus, the methods are only briefly described in an appendix to the report. This is often the case with large reports with little opportunity for reflection on the methods used. The purpose of this paper, therefore, was to discuss contributions of this review to developing methods for involving end‐users in the production of systematic reviews. We also reflect on the impact of end‐user involvement on our evidence synthesis and highlight some of the issues and challenges faced.

Despite the publication of several systematic reviews of patient and public involvement in research^8^, ^9^, ^10^, ^11^, ^12^, ^13^, ^14^, ^15^, discussion continues regarding methodological best practice. In particular, there is debate surrounding the most effective and appropriate methods for meaningful (rather than tokenistic) involvement, best methods to engage end‐users and to ensure that the perspectives of all relevant parties are incorporated especially those which are harder to reach, and mechanisms to ensure that the process is fruitful for all those involved. This is especially so for systematic reviews, an area of research in which there are few methodological accounts or evaluations of end‐user involvement.^2^, ^8^, ^16^, ^17^ A systematic review of public involvement in the systematic review process published in 2011 by Boote *et al*.^1^, ^16^ identified seven case examples. Within these, contributions from patients and the public had been made in five areas of the systematic review process (i) refining the scope of the review, (ii) suggesting and locating relevant literature, (iii) appraising the literature, (iv) interpreting the review findings and (v) writing up the review. Progress in this area of methodological expertise is hampered by the lack of evaluations and comparisons of different involvement strategies in terms of benefits and challenges or the impact on findings. End‐user engagement in research of all types can be time and resource intensive, and it would seem sensible to establish guidelines for best practice. The aims of this study were therefore to (i) highlight the methods of end‐user involvement used in our reviews, (ii) to facilitate and stimulate discussion of the most appropriate and efficient methods of engagement and (iii) to develop a list of suggestions to improve future involvement in systematic reviews.

## Methods

### Identification of end‐users

The project team included experts in clinical psychiatry, ADHD, paediatrics, education, qualitative and quantitative research synthesis methods and statistics. Parents of children with ADHD, special educational needs specialists and experts in developing and delivering non‐pharmacological interventions for ADHD in the school setting were also approached and asked whether they would like to be involved with the project. At a collaborative level, a parent of children with ADHD with experience of working as a teaching assistant and running a support group for parents of children with ADHD (CS) and a behavioural support professional (WP) were an integral part of the project team. These individuals were identified through personal contacts and were approached to join the team as it was felt that their multiple perspectives of lived experience of ADHD in the school setting would enhance the project. Informal training and support was provided by the research team in response to questions from end‐users as they arose. For example, explanations of research methods and terminology were provided both in written documents and during *Event 1,* and questioning and debate were encouraged. The training worked both ways; end‐users also taught researchers about aspects of ADHD and schools. In terms of support, the PenCRU Family Faculty (http://www.pencru.org/getinvolved/ourfamilyfaculty/) coordinator attended *Event 1* and was available to support and update individuals throughout the project. The research team encouraged and valued contributions made by individuals, were sensitive to the use of open and accessible language and provided opportunities to feedback on documents via the telephone rather than in writing. An Expert Advisory Group was convened that comprised individuals from an academic perspective (professors of education, social‐emotional development and child and adolescent psychiatry), a charity perspective (head of research and education at a UK children's disability charity), and an intervention perspective (developer of an intervention used to manage ADHD in schools) and were involved throughout the project in a consultative capacity. Examples of this involvement include individuals commenting on the protocol, editing draft chapters and responding to *ad hoc* questions as they arose during the project. In addition, a series of events were held during the project to engage with other end‐users on a consultative basis.

#### Event 1

A workshop during the first month of the project. Participants included parents and carers of children with ADHD (recruited from the PenCRU Family Faculty http://www.pencru.org/getinvolved/ourfamilyfaculty/), teaching professionals (recruited from existing contacts from other school‐based research projects) and researchers. The aim of the workshop was to share information about the project and to explore end‐user knowledge and experience about non‐pharmacological interventions and child outcomes in schools. The workshop began with a presentation from researchers giving a broad overview of the project and the methods due to be employed. Participants were then split into small groups according to their background, for example researchers, practitioners and parents, and asked to discuss firstly the range of non‐pharmacological interventions used in the school setting and secondly relevant outcomes that may be used to assess the effectiveness of such interventions. At the end of each discussion, the small groups fed back to the whole group to allow cross‐disciplinary dialogue, and notes were taken. A summary of the whole group discussion was sent to all participants, and we involved those who were invited but could not attend by asking for feedback on the meeting notes.

#### Event 2

**Figure 1:**
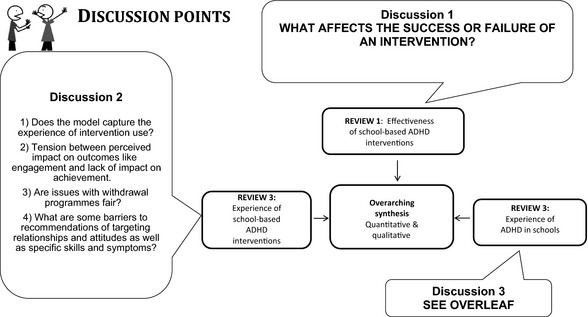
Discussion worksheet for Event 2.

**Figure 2:**
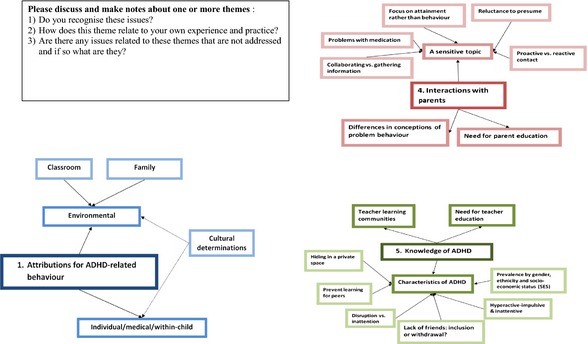
Discussion worksheet for Event 2.

A workshop after 12 months of the project with a group of behavioural support advisory teachers. Participants were colleagues of WP and were therefore identified and invited to take part in the workshop by WP. The aim of the workshop was to explore interim findings from three components of the project: a review on the effectiveness of school‐based non‐pharmacological interventions for ADHD delivered in the school setting, a review of qualitative research on the experience of these interventions in schools and a review of qualitative research on the experience of ADHD in schools. A short presentation was given by researchers for each of the reviews, and then, practitioners worked in small groups to contribute information about their experiences relevant to the review. The researchers produced a worksheet to aid and focus discussion (Figs 1 and 2).

#### Event 3

A seminar after 12 months of the project at a parent support group coffee morning with a group of parents of children and young people diagnosed with autism spectrum disorders and/or ADHD. The aim of the seminar was to explore the interim findings of the review of qualitative research on the experience of ADHD in schools. A short presentation was given by one of the research team and then parents worked in small groups to contribute information about their own experiences in relation to the findings.

#### Event 4

A 1‐day seminar was organized by the charity Cerebra. This seminar aimed to provide parent and carers, the professionals that support parents and carers, clinicians and educators with an overview of review findings. The seminar was oversubscribed and, although aimed at parents, was also attended by practitioners, clinicians and policymakers.

We also presented the findings at a variety of conferences and departmental meetings; the audience at these included trainee teachers, clinical psychiatrists and academics. These events were predominantly about sharing the findings of the reviews with less opportunity for discussion resulting in impact on the findings.

We did not plan any formal assessment of the impact of end‐user involvement on the project, the participants or those carrying out the research. However, we asked the key members of the research team (DM, MR and RGJ) and CS and WP to reflect on the end‐user involvement in the project from their individual perspectives. We were unable to approach the individuals who attended any of the events as this was a *post hoc* initiative and it was felt that too much time had elapsed.

## Results

End‐user involvement was an important feature of the project. At a collaborative level, CS and WP (and members of the Expert Advisory Group) were involved in the development of the protocol, the design of the project, organizing *Event 2* (WP) and providing feedback on the final report both through face to face meetings and via email contact. The Expert Advisory Group were involved in supporting and promoting *Event* 4, advising on project design, recommending relevant research for inclusion and providing feedback on the summary of discussions at *Event* 1 and the final report in person and via email.

*Event 1* included a total of 15 participants (three parents and carers of children with ADHD, two teachers, two child psychiatrists, two child health experts and six methodological experts). Email responses providing further feedback on the summary of the discussions were received by three members of the Expert Advisory Group; *Event 2* included approximately 20 behavioural support advisory teachers; *Event 3* included approximately 25 parents of children and young people diagnosed with autistic spectrum disorders and/or ADHD. *Event 4* was attended by approximately 60 parents, educational practitioners and policymakers in psychology and education.

In their systematic review of public involvement in systematic reviews, Boote *et al*.^2^ describe five main contributions that patients, the public and carers can make to the systematic review process: (i) refining the scope of the review, (ii) suggesting and locating relevant literature, (iii) appraising the literature, (iv) interpreting review findings and v) writing up the review. In this example, end‐users contributed to (i), (ii), (iv) and (v). End‐user involvement has the potential to impact on a project in a variety of ways; for example, it may impact on the findings of the project, on the people involved in carrying out the work and on the dissemination of the results. Due to the complex nature of the project and the many ways in which end‐users played a role, it is difficult to distinguish between the impact or contribution of end‐users who were part of the team on a collaborative basis and those who contributed on a consultative basis.

### Impact of end‐user involvement on defining the scope of the review

*Event 1* was a lively event with enthusiastic end‐users keen to engage with the project. The event resulted in the identification of over 40 non‐pharmacological interventions for ADHD used in schools. These were categorized into nine groups and were used to inform the search strategies for the reviews. Over 40 outcomes that could be used to evaluate the effectiveness of interventions were also identified. A typology of interventions and outcomes was produced and used to inform the shape of the reviews (Tables 1 and 2). Participants were not required to rank the outcomes in terms of importance, but it was clear that end‐users were interested in a wide range of possible outcomes and that there was the potential for conflict between different end‐users in terms of which outcomes might be more important. Sadly, although we looked specifically for literature that addressed many of the interventions and outcomes discussed in the meeting, we were unable to identify any. This limits the potential impact of this end‐user contribution but highlights areas for further research. The discussions both at this event and in the email feedback after the event highlighted a number of topical issues allowing the researchers to obtain a good overview of the current tensions and debates within the field. An additional benefit of this engagement event was the identification of a number of sources of potentially useful information, for example organizations and charities which might hold relevant grey literature and key authors in the field. Although the research team felt that this was a useful meeting which generated lots of useful discussion, on reflection there was recognition that the timing of the meeting (pre‐defined in the protocol) was not ideal. The meeting was held after the funding had been obtained and the protocol finalized, an earlier meeting might have had more influence on the search terms for identifying relevant literature, a meeting held slightly later might have provided a forum in which we could have raised issues we faced in data collection and analyses. A greater representation of teachers and parents in the core Expert Advisory Group may have eased some of the disjuncture at this stage.

**Table 1:** Interventions identified by end‐users during Event 1

1. Whole school initiatives Nurture groups Forest school Social and emotional aspects of learning (SEAL) Sherborne movement Thrive Incredible years Waves 1–3 intervention (provision mapping) Stepping stones (inclusion)	6. Social interventions Social skills groups Social stories The incredible 5‐point scale Peer tutoring, coaching Circle of friends
2. Additional support Private tutor 1–1 support, teaching assistant Extra time for exams, exams in separate room Summer schoolsBreakfast club, after school club ADHD champion	7. Self‐regulation Computerized attention training Neurofeedback biofeedback
3. Accommodations Place 2 be Smaller classes Indoor pass Weighted jacket, stress toy Vibration Pads Voice recognition software Break time activities	8. Alternative treatments Massage Meditation
4. Behaviour management Time out Behavioural book Praise, rewards, reward charts, token economies	9. Miscellaneous Training for teachers Physical activity
5. Parent support applied to classroom 123 Magic	

**Table 2:** Outcomes identified by end‐users at Event 1

1. Symptoms Attention Impulsivity Hyperactivity	6. Emotional functioning Enjoyment/happiness at school Depression Patience Empathy
2. School outcomes Attainment, learning Attitude, engagement Exam preparedness Exclusion Detentions Attendance	7. Behavioural issues Risk Antisocial, Crime, Bullying: bully and victim Aggression
3. Scholastic behaviours Focus Disruptiveness On‐taskness, concentration Task completion Reduction in ‘out of seat’ behaviour	8. General functioning Quality of life (Coghill) Personal and life skills Activities, hobbies Creativeness
4. Social functioning/relationships Social relationships, friends, intimate relationships Relationships with adults and peers Effect on peers, parents, siblings Family functioning Reduced stigmatism Increased communication with and between teacher and families Cooperation	9. Health behaviours Smoking Alcohol Drug use
5. Intrapersonal Self‐efficacy Self‐esteem Self‐awareness (especially of how ADHD affects others) Confidence	10. Miscellaneous Driving (less school related)

### Impact of end‐user involvement on interpreting review findings

At *Event 2,* we discussed the emerging findings from the reviews and explored to what extent the findings were recognizable in practice. This was a constructive meeting; the end‐users already knew each other, viewed the issues from similar perspectives and were engaged with the topic which facilitated discussion.

For the review of effectiveness, we asked teachers what they believe affects the success or failure of an intervention, and these factors were considered alongside the available trial evidence when developing the moderator analysis. For the qualitative reviews, participants agreed that the interim conceptual models captured the experience of interventions for ADHD in schools, and acknowledged the tension between the perceived impact on outcomes like engagement and the lack of impact on educational attainment. Other issues that were discussed included withdrawing pupils from the classroom for interventions and barriers to interventions that address relationships and attitudes. Issues identified in the review of experience of ADHD in schools that were discussed were relationships with parents, teacher knowledge of ADHD and teacher attributions for ADHD‐related behaviour.

On the whole, participants confirmed the relevance of the interim themes of the reviews in recognizing the issues and confirming many as important, and this established the potential for transferability of the interim findings. Participants were also able to offer commentary and critique to the themes, which supported the direction of the continuing analysis.

Reflection from one of the contributors (WP), however, highlighted that although this was an interesting meeting and they were pleased to help, because we were unable to give them clear guidance on which interventions they should be using (or not using), they felt that the meeting had limited benefit for them.

Although *Event 3* involved a group of parents of children and young people with autistic spectrum disorder and/or ADHD, it became clear during the seminar that parents of children diagnosed with ADHD were in the minority. The researcher was keen to explore a number of key emerging themes from the review of experiences of ADHD in schools with the parent group; ‘mothers are silenced' and deferential and assertive forms of resistance from the reviewed studies. Whilst parents did not necessarily use the same conceptual terminology as the researchers, the experiences they described were mostly commensurate with those identified in the review. This event supported the transferability of findings from the review.

*Event 4* was a large event, supported and promoted by the charity Cerebra during which we had hoped to discuss our near‐final findings to obtain further assurance that the syntheses had external validity. However, due to time constraints, the day was structured as a series of presentations with questions from the floor in a large‐group setting. The audience were keen to ask questions to aid their understanding of the findings and seemed to view the event as an information‐gathering opportunity rather than a place for discussion. Consequently, *Event 4* was more akin to a dissemination event than an opportunity to involve end‐users in the research. However, interaction with the audience at this event has informed further work in this area.

### Impact of end‐user involvement on writing up the review

Obtaining academic and a parent viewpoint on the drafts of the report was seen as invaluable by the researchers, helping to validate and fine‐tune the conclusions and recommendations for future research in particular. It was, however, difficult to allocate time and attention to make the most of end‐user input towards the end of the project when deadlines were tight. The recognition CS showed over issues highlighted in the qualitative reviews provided additional evidence for the potential transferability of the findings, and in one of the qualitative reviews, greater attention was paid to the importance of sleep to children and young people with ADHD as a result of her input. Across studies, sleep had not emerged as a priority, but as a result of this conversation, the link between quality/amount of sleep and ADHD behaviour was included in the review.

### Impact of end‐user involvement on the people participating in the project

Reflections from the three key members of the project team (DM, RGJ and MR) and the two end‐users on the project team (CS and WP) revealed a variety of potential impacts of the end‐user involvement from their individual perspectives. Firstly, there was recognition that previous experience with end‐user involvement is likely to inform future attitude. Open exploration of the beliefs and perceptions of individuals on the project team about the potential benefits and costs of end‐user involvement at the start of the project was found to be beneficial. There was reluctance from some of the project team initially as to the utility of *Event 1* in the context of a funded project with a defined protocol. However, after the workshop, there was general consensus that the workshop had been worthwhile. Secondly, team members were frustrated that having consulted with parents and practitioners in *Event 1* about the interventions used locally and the outcomes of importance to them and their children, we were unable to find any relevant information in the literature to enable inclusion of evidence about all of them in the report. Thirdly, team members felt that within the contexts of this report, end‐user involvement was more fruitful and satisfying for the reviews of qualitative evidence than the reviews of quantitative evidence. The opportunity to share preliminary findings with end‐users and then to revisit both the synthesis and the included papers was welcomed, a process which provided a sense of confidence and belief in the findings.

### Impact of end‐user involvement on the dissemination of findings

We did not plan any formal assessment of the impact of end‐user involvement on the dissemination of the findings. In our protocol, we set out a wide‐ranging dissemination plan to include publication in peer‐reviewed journals and presentation at academic conferences in both education and mental health, presenting to voluntary agencies and support groups involved in child mental health, providing plain language summaries to organizations to inform their websites, notifying clinicians via email discussion groups and to feedback findings to government departments in both health and education. For the most part, we have achieved the objectives set out in the dissemination plan although this part of the project is still on‐going (outside of the initial funding period).

### Challenges of end‐user involvement

Although an important part of this project, the involvement of end‐users was not always easy. The following challenges were identified: (i) managing expectations, (ii) managing the intensity of emotion, (iii) ensuring that involvement is fruitful for all not just the researcher, (iv) our capacity to communicate and manage the process and (v) engendering a sense of involvement amongst end‐users.

These are discussed in more detail below.

*Managing expectations*: Common to several of the end‐user events, managing expectations and balancing the enthusiasm of end‐users with a realization of what was achievable within the project scope was difficult. As researchers, we did not always feel comfortable with this and were aware, at times, that our skills in this area may not be adequate. As identified in the reflections of the project team above, it was frustrating that the enthusiasm of parents and practitioners towards particular interventions could not be supported by evidence from the literature. Relatedly, at *Event 4*, many participants attended with the intention of finding out which interventions they should be using in their practice or requesting in their schools. However, the results of the review were not as straightforward as this, and we were not able to give them straightforward solutions. It was also apparent from the reflections of contributors to *Event 2* that although they were happy to help, because the results were not clear‐cut, the engagement was not as fruitful for them as it might have been. It was, however, extremely useful for the research team. It is not unusual for a systematic review of this size and complexity to fail to produce clear‐cut recommendations, and we had pre‐warned par‐ticipants that this was the case. The challenge for researchers is to manage expectations such that end‐users are sensitive to this and are receptive to the typically more indicative nature and implications of findings.

*Managing the intensity of emotion*: In *Event 1,* we invited different end‐users including parents, practitioners and researchers with the aim of exploring different views. The workshop was led by an experienced chair (TF), we set ground rules (e.g. to respect differing viewpoints and maintain confidentiality), and we had facilitators in each discussion group who could offer the opportunity to follow up with individuals later if necessary. We allowed plenty of time for discussion so that voices and stories could be heard and scheduled regular breaks. At all events, we offered end‐users the opportunity to continue the discussion with us at a later date if necessary. However, managing the intensity of emotion between individuals with differing viewpoints was challenging at times. In particular, there was conflict surrounding the relative importance of various outcomes to different end‐users and a suggestion of ‘blame’ both from parents and from teachers. Whilst this was a challenge, it clearly highlighted this issue for the research team in a way that might not have been possible had we held separate events for different groups of end‐users.

Another potential area in which the intensity of emotion may need to be managed carefully is in the reading of draft manuscripts. CS reflected that reading the draft chapters had been an emotional experience for her. ADHD affects many aspects of her life, with several members of her family having an ADHD diagnosis; reading about the difficulties that people with ADHD face in black and white reminded her of the costs of ADHD to her family and was painful, but nonetheless, she was pleased to be involved as the drafts held the potential to help others cope with ADHD.

*Ensuring that involvement is fruitful for all not just for the researchers*: This is linked both with managing expectations and engendering a sense of involvement amongst end‐users. Discussing elements of the end‐user involvement with both WP and CS highlighted the need to balance the relationship so that all parties consider it to be beneficial. As researchers, especially those with a tight deadline, it may be easy to extract what is needed for the project from a group of end‐users without carefully considering whether the engagement is mutually beneficial.

*Our capacity to communicate and manage the process*: As a group of predominantly methodological researchers, the core team had little prior expertise in communicating with end‐users in an evidence synthesis project. Explicit training and time to develop an on‐going relationship with end‐users would have increased the teams’ confidence in dealing with the challenges. Bridging the gaps in communication between developing and submitting an application for funding and the eventual commencement of the work is an area that we did not manage well. Recruitment of study‐specific team members who were not involved in the development of the funding bid meant that there was no on‐going relationship between the core research team members and the end‐users at the start of the project. One result of this was that CS did not know she was being asked to comment on drafts of reviews for which she was a project team member. The time gap between funding application and completion of first drafts (about 21 months), the discontinuity between contact researchers, her involvement in multiple research projects and the adoption of a project acronym following allocation of funds meant she did not connect the application she had been involved with previously with the qualitative draft reviews when asked for comments. She was happy to give comments, but was astonished to learn upon consultation for this study that she had been a named team member.

Engendering a sense of involvement amongst end‐users: We involved different groups of end‐users throughout the project rather than having one central group who were called on repeatedly. We had no dedicated team member responsible for maintaining end‐user relationships, and at the most busy times in the project timetable, there was little time to think about end‐user involvement. This limited the opportunities for collaboration, most of the involvement being consultative in nature. It was also not possible for the researchers to develop a good rapport with end‐users as the opportunities for relationship building were limited. This highlights the time and resources necessary for meaningful involvement both for the project team and the end‐users, and whilst we aimed to achieve a balance between people feeling involved and burdened by the involvement, this might have reduced the sense of being involved for some people.

## Discussion

In this paper, we describe the benefits and challenges of end‐user involvement in a large project comprising of a suite of systematic reviews of qualitative and quantitative evidence on non‐pharmacological interventions for ADHD delivered in school settings, funded in response to a call from the NIHR Health Technology Assessment programme.^7^ End‐users were involved in three of the five main areas identified by Boote *et al*.,^16^ namely suggesting and identifying literature, interpreting review findings and writing up. Involvement took a number of forms including collaboration with individuals with relevant experience on the project team and engagement events with groups of end‐users to discuss interim findings. Despite the pre‐determined nature of the project, end‐user involvement had a number of impacts on the findings, including guiding the direction of the research, contributing to a typology of interventions and outcomes, the direction of the data analysis and to the robustness of the syntheses by demonstrating the alignment of interim findings with lived experiences. Challenges included managing expectations, managing the intensity of emotions, ensuring that involvement was fruitful for all, our capacity to communicate and manage the process and engendering a sense of involvement amongst end‐users.

There are few published accounts of end‐user involvement in systematic reviews.^8^, ^16^ We therefore felt it important to highlight the methods used in our reviews to facilitate and stimulate discussion of the most appropriate and efficient methods of engagement and to ensure that methods minimize harms both to the research itself and to those involved.^18^ End‐user engagement in research of all types can be time and resource intensive and it would seem sensible to establish guidelines for best practice. The idea for this study developed during the project, and thus, there were no *a priori* plans in the protocol to assess the impact of end‐user involvement on the people involved in the project or the plans for dissemination. Similarly, we were unable to gather the views of people involved in the individual end‐user events partly because it was not an *a priori* objective for the study and partly because time and resources were focussed elsewhere. The results of the evaluation are therefore potentially limited as they only present the perspectives of the core research team and of end‐users who were embedded within that team. However, comments and opinions were elicited by one‐to‐one email and telephone calls in an attempt to encourage individuals to be frank and honest.

End‐user involvement in this project had the biggest positive impact on the researchers involved in the qualitative synthesis. Discussing interim analyses with end‐users was felt to be extremely valuable as it helped to reassure the reviewers that the emerging findings were not completely ‘out of left field’ but that aspects were recognizable or ‘struck a chord’ with readers. End‐user responses to interim findings included (i) ‘recognition’ where the end‐users described their own experiences in line with the findings of the review, (ii) ‘lack of recognition’ where the end‐users did not seem to have experience of a finding and (iii) identification of ‘gaps’ where end‐users talked about issues not brought out by the research. ‘Recognition’ helped establish the potential for transferability, ‘lack of recognition’ questioned transferability and ‘gaps’ informed us about the applicability of the review. However, we could not discuss gaps unless the pertinent data were available in included studies and relevant to our review questions (e.g. the outcomes and interventions highlighted by *Event 1* that could not be addressed as we were missing from the studies included in *Review 1* see above). Involving end‐users in this way draws from the idea of member checking, a method that has been described in relation to checking the validity of both primary qualitative research and review findings, for example consulting with people with similar experiences to those involved in primary qualitative research,^19^ checking review conclusions with the primary authors of included studies^20^ as well as consulting key informants or focus groups to check the validity of review findings.^21^ However, in consulting ‘in‐group’ stakeholders, our approach differed from member checking because the stakeholders were not those from whom the data had been collected. Rather than credibility, the purpose was to establish potential transferability, because in qualitative research, it is only the reader who is able to come to a conclusion about whether research findings are transferable to their own context. The responsibility of the researcher (or reviewer in this case) is to report findings with ‘thick description’ sufficient to allow judgements of transferability to be made by the reader.^22^

There are a multitude of different approaches that could be utilized in end‐user involvement in systematic reviews at all stages of the review process. Several authors have attempted to simplify this diversity with the use of conceptual frameworks.^11^, ^15^, ^23^ Shippee *et al*.^15^ suggest that the use of a common framework and language will help to standardize and clarify the future evidence base. Oliver *et al*.^23^ recommend the use of the framework as a basis for discussion in the design and evaluation of involvement activities. The need for quality standards for the involvement of service carers and carers in systematic reviews has been discussed previously.^16^, ^24^ Braye and Preston Shoot^24^ suggested in 2005 that quality standards should address questions such as who was involved, how did they participate, what level of involvement was offered and why, what training and support was offered, who controlled the questions to be asked and what were the outcomes of participation both for the review and for the participants themselves. The GRIPP2 checklist that aims to provide guidance to enhance the quality of patient and public involvement in research is currently being developed^25^, whilst this is not specific to either the wider end‐user involvement in our study or systematic reviews, reporting standards have been shown to have implications for the design and data collection of studies.^26^ A set of quality standards or reporting guidelines specifically tailored to the involvement of end‐users in systematic reviews would be useful to those appraising the protocols of proposed reviews (e.g. funders, Cochrane review groups, end‐users being asked to participate in reviews) and to those involved in the production of reviews (e.g. researchers and end‐users).

Based on this project, we have developed a list of suggestions (Box 1) to improve future end‐user involvement in systematic reviews, factors that we would have found valuable to discuss and consider at the outset. These suggestions are organized into three phases based on the model proposed by Shippee *et al*.^15^

Box 1 **Suggestions to improve future end‐user involvement in systematic reviews**

**Getting ready for the review**

- Encourage open exploration of the views and perceptions of the project team towards the benefits and costs of end‐user involvement in the systematic review at the outset of the project.
- Consider the timing of end‐user engagement carefully and schedule meetings/events when they are most likely to have a meaningful impact on the project.
- Develop a clear plan for end‐user involvement and a central point for recruiting end‐users, allowing sufficient time and resource to allow co‐ordination and maintenance of contact throughout the project period.
- Develop and agree clear ‘ground rules’ for meetings and events which allow the contributions of individuals to be valued and respected.
- Be clear about the potential for impact of end‐user involvement on the people involved and the findings of the systematic review to enable appropriate management of expectations.
- Consider who to approach as end‐users to ensure a breadth of practice, views and perspectives are covered. Take note of the potential for attendees to have shared experiences to ensure that people can feel comfortable talking (e.g. Are the teachers and parents from the same schools? Has the psychiatrist worked with any of the families present?)
- Allow for flexibility in approach depending on the review topic, the findings and the clarity of the key messages.

**During the review**

- llocate sufficient time and resources to allow for meaningful involvement throughout the project and include end‐user involvement processes within the project timetable.
- Consider targeting the involvement and consulting with different end‐users for different tasks/aspects of the project depending on their suitability/interests, although this needs to be balanced with ensuring that all parties are benefiting from the process.
- Consider the potential for conflict between end‐users with different perspectives when organising involvement events.
- Consider holding pre‐workshops for service users to learn about methods and discuss experiences so that they are more comfortable with ‘experts’ and can rehearse contributions.

**Getting the findings of the review to those who are able to act on them**

- Be clear about the potential for impact of end‐user involvement on the dissemination of findings to enable appropriate management of expectations of all parties involved.
- End‐user involvement may be particularly helpful in identifying gaps in the research and developing recommendations for future research. Adequate time to consider not only the potential transferability of findings but also the gaps in the research can extremely valuable.

Whilst there is evidence that end‐user involvement improves the relevance of research findings, there is little guidance on how best to carry out and resource end‐user involvement meaningfully in systematic review projects. This issue may be particularly acute for projects that are developed in response to a funding call in which there may be little opportunity for shaping the direction of the review in line with end‐user views and preferences. Additionally, many systematic reviews will be conducted by those with methodological rather than topic expertise, exacerbating issues relating to relationship building and continuity amongst groups of end‐users.

To enable an understanding of the most efficient and appropriate methods for end‐user engagement in future systematic reviews, it would be valuable for methods for the assessment of the impact of end‐user involvement to be included in the protocol. Future research could inform guidelines for best practice in this area by studying in more depth the experience of involvement, both from the perspective of the researcher and the end‐user, and to explore the most effective methods for meaningful engagement. Additionally, a requirement for research abstracts to contain a section on end‐user involvement would make it easier to locate and disseminate previous examples of good practice.

## Funding source

This paper relates to project 10/140/02: Non‐pharmacological interventions for attention deficit hyperactivity disorder (ADHD) delivered in school settings: a systematic review of quantitative and qualitative research funded by the NIHR HTA programme and was supported by the National Institute for Health Research (NIHR) Collaboration for Leadership in Applied Health Research and Care South West Peninsula at the Royal Devon and Exeter NHS Foundation Trust. The funders had no role in the design or conduct of the review, data collection, analysis, or interpretation, or approval of the manuscript. The views expressed in this article are those of the authors and not necessarily those of the National Health Service, the NIHR or the Department of Health.

## Conflict of interest

None declared.
